# Individual Variation in Social Behaviours of Male Lab-reared Prairie voles (*Microtus ochrogaster*) is Non-heritable and Weakly Associated with V1aR Density

**DOI:** 10.1038/s41598-018-19737-9

**Published:** 2018-01-23

**Authors:** Andrea R. Vogel, Heather B. Patisaul, Sheryl E. Arambula, Francesco Tiezzi, Lisa A. McGraw

**Affiliations:** 1 Department of Biological Sciences, North Carolina, USA; 2 W. M. Keck Center for Behavioral Biology, North Carolina, USA; 3 Program in Genetics, North Carolina, USA; 4 Department of Animal Science, North Carolina, USA

## Abstract

The genetic and environmental factors that contribute to pair bonding behaviour remain poorly understood. Prairie voles (*Microtus ochrogaster*) often, but not always, form stable pair bonds and present an ideal model species for investigating the genetic and environmental factors that influence monogamy. Here, we assessed variation in partner preference, a measure of pair bonding, and related social behaviours in a population of laboratory-reared prairie voles under controlled environmental conditions. We evaluated to what extent variation in these behaviours correlate with vasopressin 1a receptor (V1aR) expression in the ventral pallidum (VP) and retrosplenial cortex (RSC), and estimated the heritability of these behaviours and V1aR expression. We found substantial variation in partner preference and measures of aggression, paternal care, and anxiety-like behaviours, but no correlation between these traits. We also found variation in V1aR density in the VP and RSC can account for behavioural components of paternal care and aggression, but not in partner preference. Heritability estimates of variation in partner preference were low, yet heritability estimates for V1aR expression were high, indicating that the extensive variation in partner preference observed within this population is due largely to environmental plasticity.

## Introduction

Monogamy, defined as a mated pair that stays together through several breeding seasons, is rare among mammals, and when it occurs, monogamy is typically accompanied by the formation of pair bonds – strong, lasting social bonds between mates^1^. Prairie voles (*Microtus ochrogaster*) are socially monogamous rodents that often form pair bonds but, in their natural habitats, display great variation in their level of social monogamy^2–6^. For example, male prairie voles vary considerably in their mating strategies, ranging from “resident” strategies, where they defend a territory with their respective paired female; to “wanderer” strategies, where they do not have defined territories and gain paternity through extra-pair matings^2–5,7,8^. In addition to decades of field research describing mating system variation in their natural habitats, prairie voles have also become an invaluable laboratory species for understanding the neurobiological and genetic basis of pair bonding and related social behaviours. While these laboratory studies have increased our understanding of the biological basis of the pair bond, our understanding of why individuals show such strong variation in mating strategies, including whether or not they form pair bonds at all, is still poorly understood. In this study, we set out to further characterise natural genetic variation in male pair bonding and related social behaviours, its neurobiological biological correlates, and the heritability of these traits in a controlled laboratory environment.

Pair bonding and related social behaviours in prairie voles have been studied in the laboratory using standard rodent behavioural assays. The pair bond is commonly measured using the partner preference test, which measures the amount of time a focal animal spends with its mate (partner) versus an unrelated, unfamiliar animal of the same sex (stranger). In prairie voles, males typically form a “partner preference” whereby they spend more than twice the amount of time with their “partner” than the “stranger” female following a brief cohabitation and mating^9–11^. Pair bonding also alters related social behaviours in prairie vole males that can also be easily measured in the laboratory. Increased aggression towards a same-sex intruder is perhaps the most well-characterised post-pair bonding alteration of male behaviour and is thought to be an adaptation to territorial defense and mate guarding^12^. In addition, paternal behaviour, measured indirectly as alloparental care, and anxiety-like behaviours, measured with the open field test, are also sometimes altered following the formation of a pair bond^13,14^. The close association of pair bonding and related social behaviours is partially explained by the extensive overlap of the neural circuitry encoding these behaviours, and presumably, a common genetic basis^15^.

Several neurobiological systems underlying male pair bond formation and maintenance have implicated multiple neurotransmitters and their respective receptors as an important mediator of male social behaviours^6,10,16,17^. Most relevant to this study is the role of arginine vasopressin and its receptor (V1aR) within the ventral pallidum (VP) and the retrosplenial cortex (RSC) in mediating male social behaviours including pair bond formation, aggression, and anxiety^8,18–21^. V1aR expression within the VP shows considerable natural variation, and manipulation of the density of V1aR modulates male social behaviour^20–23^. For example, antagonists against V1aR in the VP reduce a male’s propensity for pair bonding^20^ and anxiety^21^, whereas increasing V1aR binding in this brain region increases partner preference formation and alloparental care^19^. In prairie voles studies in semi-natural environments, V1aR expression in the RSC predicts male behaviours associated with pair bonding including sexual fidelity and intrusion rate, whereby male voles who had higher levels of V1aR were more likely to only have offspring with their partner and less likely to intrude on another male’s territory^8^. Expression of V1aR in both the VP and RSC exhibits considerable natural variation, yet whether or not this variation predicts pair bonding behaviours or whether or not this expression is heritable is poorly understood^22,23^.

Although pair bonding, related social behaviours, and V1aR expression in the brain are complex traits presumably encoded by multiple genes, polymorphisms in and near *avpr1a*, the gene encoding V1aR, appear to play a significant role in modulating male social behaviour. A polymorphic microsatellite region upstream of the *avpr1a* locus has repeatedly been shown to influence several male social behaviours, including partner preference, along with paternal care and anxiety^23–25^. Congruence of these results, however, has been inconsistent and other studies did not observe correlations between *avpr1a* microsatellites and social behaviours, showing that other mechanisms are involved in complex social behaviours^7,26,27^. Even so, V1aR levels in the RSC were associated with single nucleotide polymorphisms (SNPs) in *avpr1a*, which points to a genetic basis for spatial memory and sexual fidelity^8^. Although the polymorphisms at the *avpr1a* locus undoubtedly influence both social behaviour and brain V1aR expression, studies of these polymorphisms in complex, natural or semi-natural environments attribute a smaller role to *avpr1a* polymorphisms^7,26,27^. We speculate that genetic background effects or genotype x environment interactions could be the basis for phenotypic variation in prairie voles.

Based on decades of previous research on social behaviours in these rodents, we have discovered extensive variation in social behaviours, variation in V1aR density in socially-relevant areas of the brain, and potentially functional allelic variation in *avpr1a*. Social behaviours, in general, are complex traits that arise from the influences of many genes. These behaviours have been assumed to be heritable in prairie voles, since work in other organisms has shown measurable heritability for most behavioural traits^28^. In order to test the hypothesis that genetic variation in part accounts for variation in social behaviour observed in field and laboratory populations of prairie voles, we assessed variation in male partner preference behaviour and other social behaviours in a population of laboratory reared prairie voles under carefully controlled environmental conditions. This experiment was originally designed to create a genetic mapping population for future projects. We estimated the heritability of partner preference and social behaviours, including anxiety, alloparental care (care for young that are not offspring of the experimental male), and aggression towards a same-sex intruder (a proxy for mate guarding). We also evaluated the extent to which variation in V1aR expression in the VP and RSC is correlated with behavioural variation. We discovered that heritability of the social behaviours, including partner preference, anxiety-like behaviours, alloparental care, and aggression towards a same-sex intruder, is not significantly different from zero; but that variation in vasopressin receptor expression in the VP and RSC is significantly correlated with affiliative behaviours.

## Results

### Males vary in their partner preference

**Figure 1:**
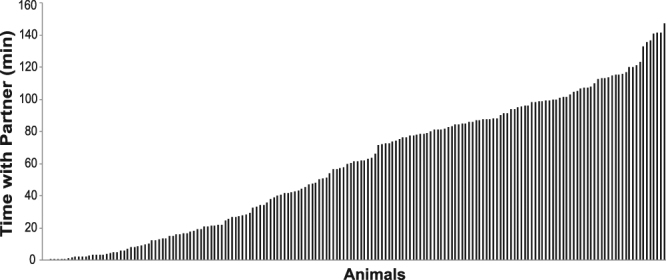
Distribution histogram of pair bond formation in an outbred laboratory population of male prairie voles.

We used a standard partner preference test to assess partner preference^9^. Briefly, the focal male’s partner was tethered at one end of a three chambered apparatus, while an unfamiliar female neither the male nor female partner had met before was tethered at the other end of the apparatus. The male was free to move around the chamber and spend time with his partner, the stranger, or by himself. We measured the time each of 180 males spent with his partner for 180 minutes. The average time (±SE) spent with the partner was 59 ± 3 min. However, we observed extensive variation in partner preference, ranging from 0 to 147 min (Fig. 1).

### Partner preference is not associated with other social behaviours

Because behaviours including aggression toward conspecifics, paternal behaviour, and anxiety-like behaviours are often influenced by pair bonding, we measured these behaviours both before males were paired and after the partner preference test. Aggression was measured using the resident intruder test where we measured latency to approach a strange male, time spent away from the strange male, affiliative behaviour towards the strange male, defensive behaviour, and aggressive behaviour. Aggressive behaviour in the resident intruder test increased dramatically after mating (Table 1). Alloparental behaviour was assessed by introducing a male to unfamiliar pups. We measured latency to approach pups, time away from the pups, licking and grooming, cuddling with the pups, carrying the pups, and aggression towards pups. Most aspects of alloparental care were unaffected, except for licking and grooming of the pups, which decreased after mating (Table 1). Further, we did not observe changes in anxiety-like behaviours as measured using the open field test. Interestingly, we did not observe a significant correlation between any of these male behaviours and partner preference (Supplementary Fig. S1), demonstrating that there is substantial individual variation in the responses of males to mating.

**Table 1:** Comparisons of time spent performing male behaviours before and after mating. All behaviours are durations measured in seconds, except for aggression during the alloparental care assay, which is measured as a frequency.

Behaviour	Before Mating (s, Mean ± SE)	After Mating (s, Mean ± SE)	P
**Alloparental Care**			
Latency to Approach	29 ± 4	21 ± 3	0.0753
Move Away	80 ± 5	80 ± 6	0.9555
Licking/Grooming	38 ± 3	30 ± 2	0.0048
Huddling/Hovering	142 ± 6	148 ± 7	0.3402
Carry Pup	4 ± 1	3 ± 1	0.2442
Aggression	0.02 ± 0.01	0.07 ± 0.01	<0.01
**Resident Intruder**			
Latency to Approach	19 ± 2	13 ± 1	<0.01
Alone	205 ± 4	229 ± 3	<0.0001
Non-aggressive	63 ± 3	27 ± 3	<0.00001
Defensive	4 ± 1	3 ± 0.5	0.0204
Aggressive	8 ± 1	28 ± 2	<0.00001
**Open Field**			
Centre	124 ± 6	113 ± 6	0.0713
Edge	779 ± 6	790 ± 6	0.0718

### V1aR density associates with some pair bonding related behaviours

**Figure 2:**
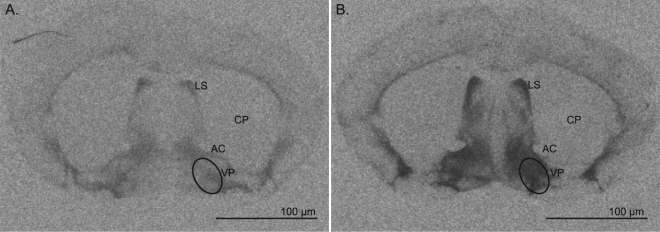
Association of variation in V1aR density in the ventral pallidum with affiliative behaviour. (**A**) A representative section showing low V1aR density in the VP (**B**) A representative section showing high V1aR density in the VP. VP, indicated in the circled region, designates ventral pallidum, AC for anterior commissure, CP for caudate putamen, LS for lateral septum. Scale bar = 100 μm.

**Figure 3:**
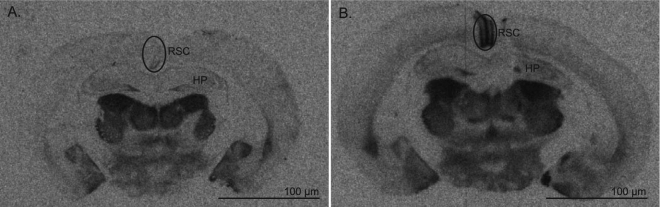
Association of variation in V1aR density in the RSC of male prairie voles with variation in affiliative behaviour. (**A**) A representative section showing low V1aR density in the RSC (**B**) A representative section showing high V1aR density in the RSC. RSC, indicated by the circled region, designates retrosplenial cortex, HP for hippocampus. Scale bar = 100 μm.

To evaluate the correlation between V1aR abundance with variation in partner preference, aggression, alloparental care, and anxiety-like behaviours, we quantified the V1aR density in the VP and RSC of male prairie voles. We collected brain tissue for V1aR assessment in a subset of tested males. Autoradiography was performed on brains collected immediately after the final behavioural test. We used rank-order Spearman correlation tests to assess variation in each behavioural component with V1aR density in each of eight rostral to caudal sections through the VP and seven sections through the rostral RSC. We observed significant variation in V1aR densities across the males both in the VP (Fig. 2A and B) and the RSC (Fig. 3A and B). After controlling for multiple comparisons, none of the correlations reached statistical significance. Importantly, however, in contrast to previous observations, we did not observe a correlation with partner preference and V1aR density in either the VP or RSC (Supplementary Tables S1 and S2). Cuddling with pups after mating was associated with four sections and the total average of V1aR density in the VP (R = 0.28–0.40; Supplementary Table S1). Similarly, we found that variation in V1aR density in the RSC was associated with time away from the pups, licking and grooming, cuddling, carrying, time away from a strange male, and affiliative behaviour toward a stranger (R = −0.43–0.42; Supplementary Table S2). The total average V1aR density in the RSC was associated with licking and grooming the pups before mating, the difference of time spent away from the pups before and after mating, and affiliative behaviour towards the intruder male before mating, after mating, and the difference between before and after mating (R = −0.40–0.38; Supplementary Table S2). Thus, our results show that variation in V1aR density in the VP and RSC correlates with the behavioural components of alloparental care and affiliative behaviour, but not in partner preference.

### Heritability of behaviour is not significantly different from zero

**Figure 4:**
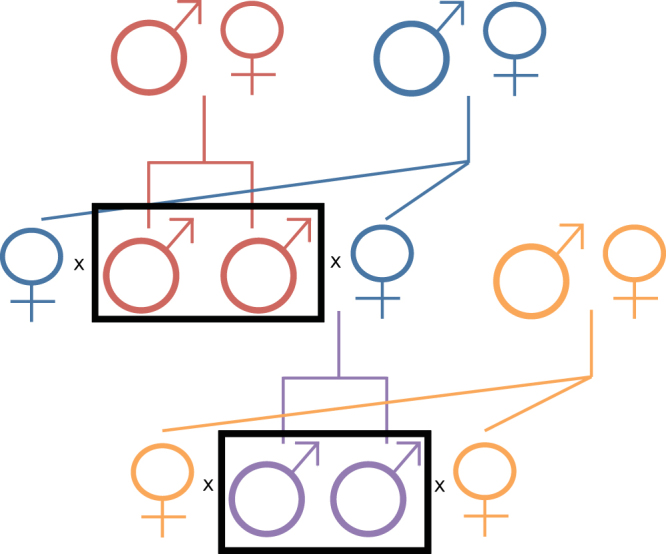
Experimental animal crossing scheme. Animals in the black boxes are the experimental males. Males were mated to unrelated full-sib females. Following behavioural testing of the parents (red male symbols), one male and one female were sacrificed and brains harvested. The remaining male and female were mated to produce the next generation offspring (purple male symbols).

The males tested for variation in partner preference and related social behaviours were derived from a crossing scheme to minimize genetic background variation. We crossed two full-sib males with two unrelated full-sib females. The male offspring of these pairings were mated to two unrelated full-sib females (Fig. 4) to generate two generations of test subjects. We measured behaviours of parents and offspring before and after mating. We were able to utilize this design to estimate the heritability of these traits. Although the laboratory population was outbred, individuals were related due to maintenance of a finite population. Therefore, we used pedigree data of all the animals in the study to derive a relationship matrix for heritability estimation. Heritability estimates of variation in the amount of time spent with the partner were not significantly different from zero (Supplementary Table S3), indicating that the extensive variation in partner preference observed within this population is largely due to environmental variation.

### Heritability of vasopressin 1a receptors is high

Using 60 of the same animals as above, we calculated heritability of density of the V1aR in the VP and RSC. Heritability estimates of the variation of the density of V1aR in the VP was 53%, while estimates in the RSC were 80%. This indicates that most of the variation seen in the density of V1aR in these regions is due to genetic variance.

## Discussion

We found substantial variation in partner preference and other social behaviours in 180 male prairie voles from a laboratory-reared population but no evidence of genetic variation for these traits. Of particular interest is the considerable variation observed in partner preference, whereby some male prairie voles spent over two hours in contact with their partner, while other males spent less than twenty minutes with their partner, during a three-hour long test. Although there was individual variation in the other behaviours we examined, there were no significant correlations between suites of behaviours. As stated in the introduction above, this experiment was originally planned to create a genetic mapping population, but due to the low heritability of the behaviours, this was inadvisable.

Our inability to detect heritability of partner preference and related behaviours may potentially be resolved by increasing the sample size to reduce standard errors and resolve a contribution of genetic variation to the phenotypic variance. Social behaviours, including partner preference, are complex behaviours that are most likely influenced by multiple genes, not all of which have been identified^29,30^. Although our population has been outbred since its arrival at North Carolina State University and DNA sequencing of a subset of BAC clones derived from this population revealed genetic variation among individuals^31^, we do not know about the historical inbreeding that may have occurred prior to establishing the population, thus weakening our ability to detect contributions of genetic variance to the social behaviours. In wild populations, approximately 2% of pairs are related to each other^16,32^. Since we did not characterise polymorphisms at the *avpr1a* locus in this population, we cannot exclude a minor contribution to the observed variation in pair bonding by variation at the microsatellite region in this gene. Although absence of polymorphic variation at the *avpr1a* locus is unlikely, it cannot be excluded.

As in previous studies, we saw considerable variation in V1aR density in the VP and RSC^22,23^. We predicted that V1aR density in these regions would correlate with social behaviours. However, variation in V1aR density did not correlate with partner preference. Our observations regarding partner preference, in some respects, do not replicate previous studies, which have shown both positive and negative relationships. For example, Hammock *et al*. reported a strong negative correlation between V1aR density and partner preference, while Barrett *et al*. reported no correlations between contact with the partner and V1aR density^21,23^. Regarding positive relationships, multiple studies demonstrate a link between partner preference and increased V1aR density in the VP^6^. Our observations regarding affiliative behaviours, such as cuddling, licking/grooming, and carrying the pup, and affiliative and defensive behaviour towards a strange male, do replicate and extend previous work from other laboratories. Our study found consistent correlations with these affiliative behaviours and V1aR density across the VP and RSC, similar to Hammock *et al*.^23^, which to our knowledge is the only other study that has performed this analysis. As shown in Table 1, most alloparental behaviours, with the exception of licking/grooming of pups, and open field behaviours did not significantly change in duration when measured before and after mating. All of the behaviours measured in the resident intruder test did change in duration after mating, compared to before mating. The direction of change can be seen in Table 1. While we cannot say for certain why V1aR expression is associated with some behaviours before mating, but not after, we speculate that the changes in environment (female, mating, pair bonding, etc.) are driving the changes in behaviour. Therefore, we would argue that mating experience does influence those behaviours. For certain, aggression towards a same-sex intruder has been well documented in other laboratories to increase after mating. According to Terleph *et al*., contact with a female will increase responsiveness to a pup, even without mating^13^. These complex findings beg the question of why our study replicates the associations between V1aR density and affiliative behaviour, but not partner preference. One possibility is a technical difference in how we assessed partner preference. In this study, we assessed partner preference as the total time spent in contact with the partner, and like a study from a different laboratory that assessed partner preference the same way, we found no correlations between V1aR density and partner preference^21^. Previous studies assessed partner preference as spending more than twice as much time with the partner as with the stranger^19^. We chose this method because it is a direct measurement of the actual partner preference behaviour. However, our data show that there is no correlation between these social behaviours and partner preference based on differences in V1aR expression.

Despite the controlled environment under which these studies were performed, the substantial variation in social behaviours that we observed, as well as our inability to detect heritability of these traits suggests a strong environmental component to pair bond formation. In population and quantitative genetics, variance in a phenotype, such as behaviour, can be made up of genetic variance and environmental variance (in its simplest form of the equation). In this study, we found narrow-sense heritability, which is a sub-division of genetic variance, and can be calculated as the ratio of direct additive genetic variance to phenotypic variance. Anything that is not genetic variance is grouped into the environmental variance. This broad category can include maternal effects, toxicants, or social environment, to name just a few. Genetic and neurobiological studies of prairie voles in naturalistic environments support our conclusions that environmental variance may play a larger role in expression of social behaviours than genetic variance. For example, space use, such as territory size and whether or not a male wanders off territory, was a greater correlate of *avpr1a* microsatellite polymorphisms and SNPs at the *avpr1a* locus and V1aR density at the RSC than pair bonding^7,8^. Although we were able to carefully control the laboratory environment in which these experiments were performed, we are unable to exclude the role of the social environment in influencing pair bonding-related behaviours in this study. For example, our study confined animals to standard laboratory caging, limiting the male’s choice of mating strategy by only providing him with one possible mate. It is widely accepted that females play an equally important role in mating, yet this study did not account for female choice of mates or other male x female genotypic or phenotypic interactions.

We found heritability of V1aR density in the VP and the RSC to be 53% and 80%, respectively. The high heritability of V1aR density, but the low heritability of behaviour, is in line with work done by Perkeybile *et al*., who cross-fostered prairie vole pups and found alloparental care behaviours were more correlated with the behaviour of the foster parents, but V1aR and oxytocin receptor (OTR) densities were correlated in a sex-specific manner with the birth parents^33^. This supports our idea that environment is a more important factor for complex behaviours such as pro-social behaviours than receptor density in behaviourally-relevant brain areas.

In summary, we found substantial variation and non-significant heritability of behaviours related to monogamy among prairie voles, suggestive of a strong role for environmental plasticity of pair bonding-related behaviours and its neurobiological correlates. Future studies of prairie vole behaviour both in the laboratory and in natural populations will continue to resolve the complexity of social behaviours and help resolve the role of the environment in influencing the genetic and neurobiological underpinnings of these traits.

## Materials and Methods

### Animals

All animals were 4–7 generations of a laboratory-bred colony at NCSU, since starting in 2011. Animals were brought from the Young lab at Emory University, although originally derived from wild-caught individuals from Illinois and were interbred with wild-caught Illinois prairie voles in 2008. They were reared in house and housed in single-sex 0.3 × 0.3 × 0.2 m cages containing two to four individuals at the Biological Resources Facility at North Carolina State University (72 °F, 30% average relative humidity). Food and water were provided *ad libitum*, with corncob bedding (Anderson Bed-o-cob, Granville Milling Co., Creedmoor, NC) and paper strips for nesting material. All rooms were on a 12-hour light/dark schedule (6AM-6PM lights on). Experimental animals were eight to twelve weeks old and weighed between 30–80 grams. Experimental protocols were approved by and performed in accordance with the North Carolina State University Institutional Animal Care and Use Committee (IACUC) and the resident veterinarian.

### Mating

We injected female prairie voles with 0.1 mL of 20 μg/mL estradiol benzoate (Fisher BioReagents) once a day for the two days prior to mating in order to induce ovulation and receptivity towards males, a commonly used method^34,35^. We then paired the females with a sexually naïve male for 18 hours, which is long enough for a pair bond to form^18^. After mating, paired males and females were housed together.

### Behavioural assays

We subjected sexually naïve male prairie voles to three subsequent behavioural tests (open field test, alloparental care test, and resident intruder test), described in detail below. These tests were performed on the same day, and in the order listed above for all animals. Following the behavioural assays, we paired the males with an unrelated, sexually naïve female. After an 18-hour cohabitation period, males were tested in the partner preference test. The open field test, alloparental care test, and the resident intruder tests were repeated on the next day. The total sample size consisted of 180 males. If an animal or its mate died during the procedure, both sibling experimental males were removed from analyses.

#### Partner preference test

The partner preference test measures social preferences^9,11,36^. In this test, the partner female and a stranger female were tethered at opposite ends of a 0.6 × 0.15 × 0.3 m box. The male was introduced in the middle of the cage and interactions were recorded for 3 hours. Testing was done in the morning and afternoon, at 8:00 AM and 12:00 PM, respectively. We used the same females for both sessions, and each male was tested in a single session. Each female acted as the partner in one session, and the stranger in the other session, to match for sexual experience. Between testing sessions, the corncob bedding was removed and the arena cleaned with 70% isopropyl alcohol. All tests were video recorded and scored using TopScan (version 3.00), as previously described^11^. We recorded the time each male spent alone, in social contact with his partner, or in social contact with the stranger female. At the end of the test, each male was returned to his cage with his partner female.

#### Open field test

The open field test evaluates anxiety-like behaviour^37^. A single male vole was placed in an empty 0.6 × 0.6 × 0.6 m box. The vole was allowed to move naturally and was videotaped for 15 min, starting immediately when it was placed into the testing arena. All testing was done under overhead illumination, between the hours of 8:30 and 11:30 AM. We analysed the videos using TopScan (Clever Sys Inc., version 3.00, Reston, VA, 2011). Aggregated times spent in the centre or the edge were used for statistical analyses. The edge was defined as the area 0.1 m from the edge of the box on all sides, and the centre was defined as the remaining inner area.

#### Alloparental care

The alloparental care assay evaluates parental behaviour towards an unfamiliar pup^38^. A single male vole was exposed to two 1–4 day old pups in a 0.3 × 0.3 × 0.2 m box with corncob bedding. The experimental vole had 5 min to interact with the pup unless the adult displayed aggressive behaviour, at which point the test ended. All testing was done under overhead illumination between the hours of 8:30 and 11:30 AM. Behaviour was videotaped and we used Stopwatch+ (version 1.5.1, 2003) to quantify the latency of time the animal took to approach the pup and the amount of time spent away from the pup, huddling/hovering over the pup, licking/grooming the pup, and carrying the pup^39^. Aggressive behaviour was analysed in a binary format: 0 for no aggression, 1 for aggression.

#### Resident intruder test

The resident intruder assay measures aggressive behaviours toward an unfamiliar, unrelated male^40^. The experimental male was placed in a 0.5 × 0.15 × 0.2 m box with corncob bedding. After 1 min the “intruder”, a sexually naive animal of the same sex, was placed into the box and interactions were video recorded for 5 min. The weights of both the experimental and “intruder” animals were recorded before testing and did not influence the outcome of the behavioural assays. After testing both animals were returned to their respective home cages. All testing was done under overhead illumination and between the hours of 8:30 and 11:30 AM. We analysed the videos using Stopwatch (version 1.5.1, 2003) to determine the latency of time the experimental animal took to approach the intruder, the amount of time spent alone, and the amount of time the experimental animal displayed affiliative, defensive, or aggressive behaviour towards the intruder. Affiliative behaviour towards the intruder included actions such as huddling and ano-gentital sniffing, while defensive behaviour included actions such as being chased or attacked by the intruder male, and aggressive behaviours were actions such as chasing or attacking the intruder male.

### Autoradiography

We chose 60 males from across the partner preference distribution to examine V1aR density in the VP and RSC through autoradiography. The males were chosen by limiting the selection to those males whose brains were harvested immediately after behavioural testing, and then arranging them by the amount of time they spent with their partner during the partner preference test from lowest to highest. Every other male was chosen to make sure we had equal representation across the partner preference distribution. Brains were removed from males at the end of the behavioural assays and flash-frozen on dry ice, then stored at −80 °C. The order of the brains to be sliced was chosen by a random number generator. Six sets of 20 μm thick coronal slices from the medial geniculate to the front of the ventral pallidum at 120 μm intervals were mounted on Superfrost Plus slides (Fisher Scientific) and stored at −80 °C. To visualize and quantify V1aR binding we used standard protocols for receptor autoradiography by using ^125^I-labeled linear vasopressin V1a receptor ligand ([^125^I]-Phenylacetyl-D-Tyr(M)-Phe-Gln-Asn-Arg-Pro-Arg-Tyr-NH2, PerkinElmer Scientific [NEX310]^41,42^. Briefly, sections were lightly fixed in 0.1% paraformaldehyde in phosphate-buffered saline (pH 7.2) for 2 min at room temperature, then washed twice for 10 min in 50 mM Tris-HCl (pH 7.4). Slides were incubated at room temperature for 60 min in 50 pM ^125^I-antagonist in 50 mM Tris-HCl (pH 7.4) with 10 mM MgCl_2_ and 0.1% bovine serum albumin (radioimmunoassay grade, Sigma, St. Louis, MO). All the slices for a specific region were incubated for autoradiography using the same batch of buffer solution. Unbound ligand was removed by four washes in 50 mM Tris-HCl (pH 7.4), 10 mM MgCl_2_ buffer, and sections were air-dried. Sections were exposed to BioMax MR film (Kodak, Rochester, NY) for either 11 (VP) or 14 (RSC) days alongside radioactive standards (American Radiolabeled Chemicals, Inc.). Neuroanatomic boundaries were defined using two rat brain atlases^43,44^ and a mouse brain atlas^45^. High expression of V1aR results in high binding of radioactive ligand and can be measured by the optical density of film exposed to the tissue sections. We investigated VP and RSC V1aR by digitizing films and quantifying the standardized scans using MCID software. We examined 8 slices of the VP, spanning the entire region, and 7 slices of the RSC, spanning its rostral area. We estimated nonspecific binding from background levels of binding in the caudate putamen for the VP slices, and the stria terminalis for the RSC slices.

### Statistical analyses

A paired *t*-test was used to determine if male open field behaviour, alloparental care, or aggression in the resident intruder assay changed after mating. Significance was set at α = 0.004, after adjusting for multiple comparisons correction using the Bonferroni equation. To assess correlations between partner preference and male behaviours, we used the duration of each behaviour (from the open field, alloparental care, and resident intruder assays) before and after mating and the difference between the two and plotted these values against the time an experimental male spent with his partner during the partner preference test. Adjusted R^2^ and *P*-values were calculated for each scatterplot. Significance for the regressions was set at α = 0.001, after adjusting for multiple comparisons correction using the Bonferroni equation. All statistical analyses were performed in R (version 3.0.1, 2012).

#### Estimates of correlations between V1aR densities and behaviours

We used a Spearman’s rank correlation test to determine significant correlations between V1aR densities and the duration of behaviours. V1aR density in the focal region of each brain section was correlated separately with all behaviours. A P-value and a correlation value (R) were calculated for each correlation in R (version 3.0.1, 2012). After adjusting for multiple comparison using the Bonferroni equation, significance was set at α = 7.5 × 10^−5^.

#### Estimates of heritability

The narrow-sense heritability was calculated using parent-offspring regressions, but was not used due to only measuring the father’s behaviour, which gave small heritability values and large significant errors (data not shown). Therefore, a different way of measuring narrow-sense heritability was devised, that used a relationship matrix of the entire colony from its inception (N = 368). Using the relationship matrix, the degree of relatedness of any prairie vole in our colony to another prairie vole in the colony could be calculated. Therefore, this allowed narrow-sense heritability to be calculated while taking into account how related the experimental animals were to each other, which gave us a more accurate narrow-sense heritability for each chosen behaviour. We chose the behaviour that showed the most significant change from before mating to after mating from each behavioural test, and calculated the heritability of that behaviour before mating and after mating. The exact methodology for this calculation is below.

Heritabilities for all the traits were analysed using univariate linear analyses according to the following models:

1 $${\rm{y}}=1{\rm{\beta }}+{{\bf{Z}}}_{{\rm{m}}}{\rm{m}}+{{\bf{Z}}}_{{\rm{p}}}{\rm{p}}+{{\bf{Z}}}_{{\rm{s}}}{\rm{s}}+{\rm{e}}$$

where y is the vector of observations; β is the vector of systematic effects (intercept); m is the vector of random additive genetic effect of the male; p is the vector of random additive genetic effect of the partner; s is the vector of random additive genetic effect of the stranger; e is the vector of random residuals; 1 is a vector containing ‘1’ entries and length equal to the number of records, and **Z**_m_, **Z**_p_, and **Z**_s_ are incidence matrices relating the corresponding effects to the dependent variable. Vector of solutions random additive genetic effects were assumed normally distributed with null mean and variance equal to the effect estimated variance, respectively m ~*N*(0, **A**σ^2^_**m**_), p ~ *N*(0, **A**σ^2^_p_) and **s** ~ *N*(0, **A**σ^2^_s_), where **A** is a pedigree-derived relationship matrix^46^. Residuals were assumed uncorrelated e ~ *N*(0, **I**σ^2^_e_), where **I** is an identity matrix and σ^2^_e_ is the residual variance.

Variance components σ^2^_m_, σ^2^_p_, σ^2^_s_ and σ^2^_e_ were estimated in a Bayesian framework using the Gibbs sampling algorithm as implemented in the MCMCglmm R package^47^. Chains of 280,000 iterations were run, removing the first 30,000 iterations as burn-in and storing 1 sample every 25 iterations, leaving 10,000 samples for inference. Convergence was assessed by visual inspection of trace plots, number of effective samples and autocorrelation among subsequent samples, computed using functions that are built into the package MCMCglmm. The posterior mean of the saved samples was used as estimate of the parameter, standard deviation was computed and used as standard error of the estimates.

Total phenotypic variance was computed as σ^2^_m_ + σ^2^_p_ + σ^2^_s_ + σ^2^_e_, heritability for each additive genetic effect (male, partner and stranger) was computed as the ratio of the estimate for that variance on total phenotypic variance.

### Data availability statement

The datasets generated during and/or analysed during the current study are publicly available from at https://osf.io/hsjv7 and our protocols are publicly available at https://www.protocols.io/researchers/andrea-vogel.

## Electronic supplementary material

Supplementary Information
