# Supplementary material for: Individual Variation in Social Behaviours of Male Lab-reared Prairie voles (Microtus ochrogaster) is Non-heritable and Weakly Associated with V1aR Density
Source: Sci Rep. 2018 Jan 23;8:1396. doi: 10.1038/s41598-018-19737-9 (PMC5780391; doi:10.1038/s41598-018-19737-9)
Supplement: Supplementary file 1 — Supplementary Information [file 41598_2018_19737_MOESM1_ESM.pdf]

Individual Variation in Social Behaviours of Male Lab-reared Prairie voles (*Microtus ochrogaster*) is Non-heritable and Weakly Associated with V1aR Density

Andrea R. Vogel[1,2,3,*], Heather B. Patisaul[1,2], Sheryl E. Arambula[1,2], Francesco Tiezzi[3,4] and Lisa A. McGraw[1,2,3]

[1] Department of Biological Sciences, [2] W. M. Keck Center for Behavioral Biology, [3]Program in Genetics, and [4]Department of Animal Science

* Address correspondence to Andrea Vogel, email: arvogel@ncsu.edu

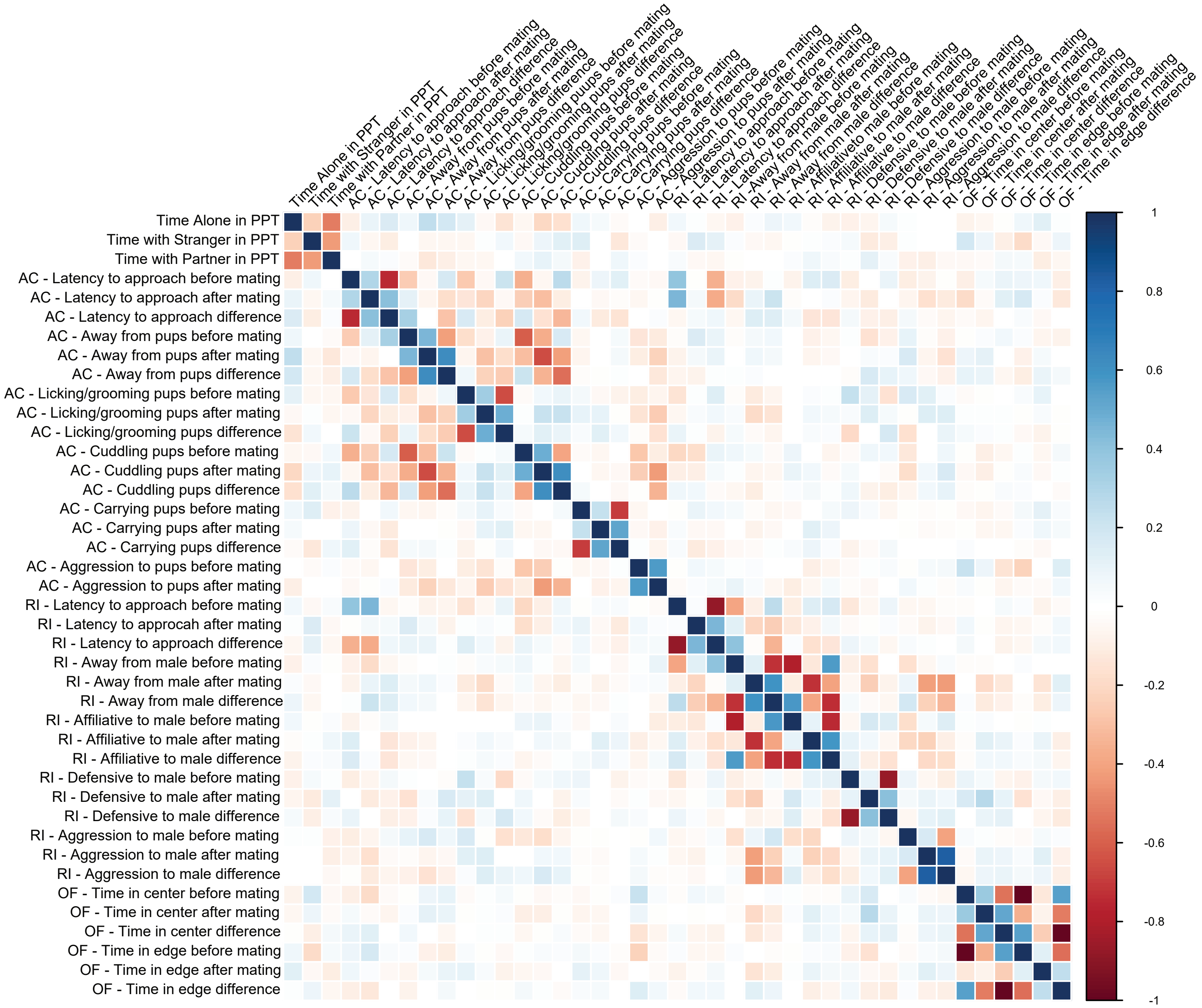

Supplementary Figure S1. Correlations of social behaviors in male prairie voles. AC designates alloparental care test, RI for resident intruder test, and OF for open field test.

Supplementary Table S1. Spearman’s rank correlation tests between social behaviors and V1aR density in sections through the ventral pallidum.

	VP Sect 1		VP Sect 2		VP Sect 3		VP Sect 4		VP Sect 5		VP Sect 6		VP Sect 7		VP Sect 8		VP Ave	
Behaviors	P	R	P	R	P	R	P	R	P	R	P	R	P	R	P	R	P	R
Time spent with partner in partner preference test	0.117	0.22	0.072	0.24	0.845	-0.03	0.822	0.03	0.082	0.23	0.136	0.20	0.060	0.24	0.597	0.07	0.075	0.23
Latency to approach pup before mating	0.875	0.02	0.783	0.04	0.177	0.18	0.258	0.15	0.912	-0.01	0.996	0.00	0.349	0.12	0.208	0.16	0.514	0.09
Latency to approach pup after mating	0.099	-0.23	0.182	-0.18	0.554	-0.08	0.705	0.05	0.109	-0.21	0.346	-0.12	0.861	0.02	0.926	-0.01	0.392	-0.11
Latency to approach pup difference	0.202	-0.18	0.210	-0.17	0.144	-0.19	0.452	-0.10	0.207	-0.17	0.347	-0.12	0.351	-0.12	0.293	-0.14	0.184	-0.17
Time away from pup before mating	0.256	-0.16	0.120	-0.21	0.407	-0.11	0.544	-0.08	0.867	-0.02	0.745	0.04	0.802	-0.03	0.367	0.12	0.572	-0.07
Time away from pup after mating	0.489	-0.10	0.206	-0.17	0.822	-0.03	0.539	-0.08	0.364	-0.12	0.969	0.01	0.773	-0.04	0.748	0.04	0.795	-0.03
Time away from pup difference	0.717	0.05	0.992	0.00	0.619	0.07	0.913	0.01	0.476	-0.09	0.745	-0.04	0.628	-0.06	0.702	-0.05	0.890	0.02
Licking/grooming pup before mating	0.185	0.18	0.121	0.21	0.770	-0.04	0.615	0.07	0.025	0.29	0.117	0.21	0.280	0.14	0.389	0.11	0.193	0.17
Licking/grooming pup after mating	0.342	0.13	0.038	0.27	0.152	0.19	0.839	0.03	0.179	0.18	0.069	0.24	0.129	0.20	0.560	0.08	0.081	0.23
Licking/grooming pup difference	0.586	-0.08	0.741	-0.04	0.211	0.17	0.820	-0.03	0.457	-0.10	0.748	-0.04	0.989	0.00	0.648	-0.06	0.976	0.00
Cuddling pups before mating	0.029	0.30	0.160	0.19	0.504	0.09	0.514	0.09	0.025	0.29	0.565	0.08	0.690	0.05	0.911	0.01	0.148	0.19
Cuddling pups after mating	0.024	0.31	0.002	0.40	0.348	0.13	0.547	0.08	0.007	0.35	0.047	0.26	0.128	0.20	0.799	0.03	0.030	0.28
Cuddling pups difference	0.790	0.04	0.069	0.24	0.518	0.09	0.689	0.05	0.205	0.17	0.055	0.25	0.077	0.23	0.422	0.11	0.168	0.18
Carrying pups before mating	0.723	-0.05	0.539	0.08	0.331	-0.13	0.348	-0.13	0.734	-0.05	0.276	0.14	0.963	0.01	0.080	-0.23	0.643	-0.06
Carrying pups after mating	0.668	0.06	0.923	0.01	0.154	0.19	0.445	-0.10	0.336	-0.13	0.827	0.03	0.379	0.12	0.384	-0.11	0.685	0.05
Carrying pups difference	0.597	0.07	0.626	-0.07	0.029	0.29	0.515	0.09	0.530	-0.08	0.375	-0.12	0.602	0.07	0.339	0.13	0.452	0.10
Aggression towards pups before mating	0.477	-0.10	0.837	-0.03	0.976	0.00	0.791	0.04	0.322	-0.13	0.954	0.01	0.977	0.00	0.887	0.02	0.711	-0.05
Aggression towards pups after mating	0.125	-0.21	0.179	-0.18	0.504	-0.09	0.928	-0.01	0.531	-0.08	0.282	-0.14	0.251	-0.15	0.815	-0.03	0.166	-0.18
Latency to approach strange male before mating	0.416	-0.11	0.854	-0.02	0.141	-0.20	0.883	-0.02	0.269	-0.15	0.339	-0.13	0.566	-0.08	0.955	-0.01	0.328	-0.13
Latency to approach strange male after mating	0.182	-0.19	0.495	-0.09	0.734	-0.05	0.773	0.04	0.533	-0.08	0.501	-0.09	0.256	-0.15	0.544	-0.08	0.310	-0.13
Latency to approach	0.706	-0.05	0.667	-0.06	0.420	0.11	0.869	-0.02	0.839	0.03	0.735	0.05	0.507	-0.09	0.338	-0.13	0.794	-0.03

strange male difference																		
Time away from strange male before mating	0.736	-0.05	0.812	0.03	0.116	0.21	0.421	-0.11	0.446	-0.10	0.913	-0.01	0.817	0.03	0.386	-0.11	0.928	-0.01
Time away from strange male after mating	0.697	-0.05	0.664	0.06	0.220	0.16	0.651	-0.06	0.194	-0.17	0.742	-0.04	0.538	0.08	0.886	-0.02	0.898	0.02
Time away from strange male difference	0.966	0.01	0.995	0.00	0.955	0.01	0.542	0.08	0.576	-0.07	0.629	-0.06	0.972	0.00	0.548	0.08	0.960	0.01
Affiliative behavior towards strange male before mating	0.859	0.02	0.945	-0.01	0.228	-0.16	0.580	0.07	0.211	0.17	0.419	0.11	0.898	-0.02	0.689	0.05	0.818	0.03
Affiliative behavior towards strange male after mating	0.485	0.10	0.921	-0.01	0.832	-0.03	0.474	0.10	0.656	0.06	0.640	-0.06	0.987	0.00	0.845	0.03	0.847	0.03
Affiliative behavior towards strange male difference	0.370	0.13	0.695	0.05	0.337	0.13	0.989	0.00	0.930	0.01	0.671	-0.06	0.609	0.07	0.807	-0.03	0.712	0.05
Defensive behavior towards strange male before mating	0.365	0.13	0.260	0.15	0.893	-0.02	0.539	0.08	0.389	0.11	0.473	0.10	0.996	0.00	0.032	0.28	0.306	0.13
Defensive behavior towards strange male after mating	0.802	-0.04	0.751	0.04	0.243	-0.16	0.377	-0.12	0.955	-0.01	0.368	0.12	0.289	-0.14	0.752	-0.04	0.492	-0.09
Defensive behavior towards strange male difference	0.580	-0.08	0.312	-0.13	0.395	-0.11	0.620	-0.07	0.729	-0.05	0.776	-0.04	0.337	-0.13	0.228	-0.16	0.367	-0.12
Aggression towards strange male before mating	0.401	0.12	0.382	0.12	0.639	0.06	0.741	0.04	0.204	0.17	0.185	0.17	0.610	0.07	0.710	0.05	0.326	0.13
Aggression towards strange male after mating	0.638	-0.07	0.961	-0.01	0.351	-0.12	0.829	-0.03	0.621	0.07	0.275	0.14	0.723	-0.05	0.280	-0.14	0.637	-0.06
Aggression towards strange male difference	0.418	-0.11	0.697	-0.05	0.321	-0.13	0.843	-0.03	0.882	0.02	0.472	0.10	0.891	-0.02	0.566	-0.08	0.591	-0.07
Time spent in center before mating	0.354	0.13	0.980	0.00	0.289	-0.14	0.883	0.02	0.717	0.05	0.500	0.09	0.980	0.00	0.199	0.17	0.788	0.04
Time spent in center after mating	0.257	0.16	0.682	-0.06	0.806	-0.03	0.987	0.00	0.833	0.03	0.670	0.06	0.755	0.04	0.251	0.15	0.749	0.04
Time spent in center difference	0.789	0.04	0.328	-0.13	0.793	0.04	0.745	-0.04	0.607	0.07	0.884	0.02	0.985	0.00	0.893	0.02	0.962	-0.01
Time spent in edge before mating	0.348	-0.13	0.974	0.00	0.313	0.13	0.874	-0.02	0.730	-0.05	0.500	-0.09	0.946	-0.01	0.196	-0.17	0.767	-0.04
Time spent in edge after mating	0.273	-0.16	0.641	0.06	0.772	0.04	0.994	0.00	0.899	-0.02	0.722	-0.05	0.756	-0.04	0.258	-0.15	0.772	-0.04
Time spent in edge difference	0.775	-0.04	0.309	0.14	0.792	-0.04	0.739	0.05	0.642	-0.06	0.891	-0.02	0.930	-0.01	0.912	-0.01	0.963	0.01

Highlighted cells have P values ≤0.05, although statistical significance is set at $\alpha = 7.5 \times 10^{-5}$.

Supplementary Table S2. Spearman's rank correlation tests between social behaviors and V1aR density in sections through the rostral part of the retrosplenial cortex.

	RSC Sect 1		RSC Sect 2		RSC Sect 3		RSC Sect 4		RSC Sect 5		RSC Sect 6		RSC Sect 7		RSC Ave	
Behaviors	P	R	P	R	P	R	P	R	P	R	P	R	P	R	P	R
Time spent with partner in partner preference test	0.399	0.11	0.972	0.00	0.978	0.00	0.486	0.10	0.193	0.17	0.383	0.12	0.279	0.15	0.328	0.13
Latency to approach pup before mating	0.987	0.00	0.708	0.05	0.943	-0.01	0.681	-0.06	0.645	-0.06	0.951	-0.01	0.642	-0.06	0.972	0.00
Latency to approach pup after mating	0.762	0.04	0.920	-0.01	0.826	-0.03	0.747	0.04	0.923	-0.01	0.894	0.02	0.665	-0.06	0.957	0.01
Latency to approach pup difference	0.562	0.08	0.980	0.00	0.920	0.01	0.441	0.11	0.607	0.07	0.721	0.05	0.785	0.04	0.827	0.03
Time away from pup before mating	0.115	0.21	0.590	0.07	0.710	0.05	0.286	0.15	0.074	0.24	0.080	0.23	0.043	0.27	0.161	0.18
Time away from pup after mating	0.225	-0.16	0.271	-0.15	0.170	-0.19	0.110	-0.22	0.126	-0.20	0.110	-0.21	0.510	-0.09	0.142	-0.19
Time away from pup difference	0.002	-0.40	0.084	-0.23	0.038	-0.28	0.006	-0.36	0.001	-0.41	0.001	-0.42	0.007	-0.35	0.003	-0.38
Licking/grooming pup before mating	0.007	0.36	0.001	0.42	0.008	0.35	0.028	0.30	0.020	0.30	0.010	0.33	0.032	0.28	0.005	0.36
Licking/grooming pup after mating	0.415	0.11	0.071	0.24	0.063	0.25	0.602	0.07	0.184	0.18	0.180	0.18	0.043	0.27	0.122	0.20
Licking/grooming pup difference	0.205	-0.17	0.317	-0.14	0.479	-0.10	0.303	-0.14	0.422	-0.11	0.386	-0.12	0.695	-0.05	0.326	-0.13
Cuddling pups before mating	0.636	-0.06	0.708	-0.05	0.939	-0.01	0.888	-0.02	0.502	-0.09	0.507	-0.09	0.369	-0.12	0.577	-0.07
Cuddling pups after mating	0.445	0.10	0.399	0.11	0.323	0.13	0.173	0.19	0.080	0.23	0.265	0.15	0.122	0.21	0.175	0.18
Cuddling pups difference	0.176	0.18	0.168	0.19	0.211	0.17	0.168	0.19	0.055	0.25	0.103	0.22	0.025	0.30	0.074	0.23
Carrying pups before mating	0.337	0.13	0.035	0.28	0.105	0.22	0.363	0.13	0.079	0.23	0.026	0.29	0.142	0.20	0.057	0.25
Carrying pups after mating	0.777	0.04	0.583	0.07	0.864	0.02	0.777	-0.04	0.948	0.01	0.780	0.04	0.853	0.03	0.846	0.03
Carrying pups difference	0.646	-0.06	0.184	-0.18	0.131	-0.20	0.179	-0.18	0.104	-0.22	0.147	-0.19	0.318	-0.13	0.154	-0.19
Aggression towards pups before mating	0.810	-0.03	0.784	0.04	0.879	-0.02	0.663	-0.06	0.535	-0.08	0.658	-0.06	0.400	-0.11	0.643	-0.06
Aggression towards pups after mating	0.975	0.00	0.826	0.03	0.975	0.00	1.000	0.00	0.832	-0.03	1.000	0.00	0.192	-0.18	0.766	-0.04
Latency to approach strange male before mating	0.632	0.06	0.847	0.03	0.233	0.16	0.340	0.13	0.722	0.05	0.662	0.06	0.267	0.15	0.467	0.10
Latency to approach strange male after mating	0.801	0.03	0.875	0.02	0.246	0.16	0.471	0.10	0.383	0.12	0.477	0.10	0.109	0.21	0.320	0.13
Latency to approach strange male difference	0.756	-0.04	0.936	0.01	0.876	-0.02	0.577	-0.08	0.733	0.05	0.826	0.03	0.945	0.01	0.897	0.02
Time away from strange male before mating	0.119	-0.21	0.458	-0.10	0.320	-0.14	0.198	-0.18	0.154	-0.19	0.129	-0.20	0.030	-0.29	0.122	-0.20
Time away from strange male after mating	0.223	0.16	0.501	0.09	0.357	0.13	0.715	0.05	0.449	0.10	0.427	0.11	0.781	0.04	0.411	0.11

Time away from strange male difference	0.112	0.21	0.419	0.11	0.345	0.13	0.448	0.10	0.195	0.17	0.198	0.17	0.199	0.17	0.180	0.18
Affiliative behavior towards strange male before mating	0.007	0.35	0.109	0.22	0.111	0.22	0.029	0.29	0.018	0.31	0.024	0.30	0.009	0.34	0.017	0.31
Affiliative behavior towards strange male after mating	0.016	-0.32	0.101	-0.22	0.029	-0.29	0.077	-0.24	0.019	-0.31	0.062	-0.25	0.168	-0.19	0.019	-0.30
Affiliative behavior towards strange male difference	0.001	-0.43	0.027	-0.29	0.015	-0.32	0.008	-0.36	0.002	-0.39	0.007	-0.35	0.005	-0.36	0.002	-0.40
Defensive behavior towards strange male before mating	0.761	-0.04	0.552	0.08	0.838	0.03	0.416	-0.11	0.619	-0.07	0.964	0.01	0.828	0.03	0.729	-0.05
Defensive behavior towards strange male after mating	0.664	0.06	0.344	0.13	0.788	0.04	0.910	-0.02	0.945	0.01	0.761	0.04	0.801	0.03	0.908	0.02
Defensive behavior towards strange male difference	0.834	0.03	0.943	-0.01	0.907	-0.02	0.644	0.06	0.935	0.01	0.832	-0.03	0.798	-0.03	0.893	0.02
Aggression towards strange male before mating	0.128	-0.20	0.477	-0.10	0.321	-0.14	0.050	-0.27	0.155	-0.19	0.714	-0.05	0.805	-0.03	0.403	-0.11
Aggression towards strange male after mating	0.716	0.05	0.328	0.13	0.249	0.16	0.379	0.12	0.254	0.15	0.589	0.07	0.421	0.11	0.316	0.13
Aggression towards strange male difference	0.528	0.09	0.355	0.13	0.269	0.15	0.161	0.19	0.168	0.18	0.527	0.08	0.369	0.12	0.237	0.16
Time spent in center before mating	0.520	0.09	0.410	0.11	0.454	0.10	0.496	0.09	0.541	0.08	0.526	0.08	0.526	0.09	0.458	0.10
Time spent in center after mating	0.852	0.03	0.537	-0.09	0.751	-0.04	0.595	-0.08	0.740	-0.05	0.489	-0.10	0.531	-0.09	0.683	-0.06
Time spent in center difference	0.673	-0.06	0.307	-0.14	0.368	-0.13	0.307	-0.14	0.382	-0.12	0.411	-0.11	0.386	-0.12	0.403	-0.11
Time spent in edge before mating	0.535	-0.08	0.435	-0.11	0.485	-0.10	0.512	-0.09	0.536	-0.08	0.543	-0.08	0.546	-0.08	0.476	-0.09
Time spent in edge after mating	0.838	-0.03	0.546	0.08	0.751	0.04	0.589	0.08	0.755	0.04	0.496	0.09	0.546	0.08	0.692	0.05
Time spent in edge difference	0.690	0.06	0.321	0.14	0.382	0.12	0.299	0.15	0.396	0.12	0.429	0.11	0.416	0.11	0.414	0.11

Highlighted cells have P values at ≤ 0.05, although statistical significance is set at $\alpha = 7.5 \times 10^{-5}$.

Supplementary Table S3. Narrow-sense heritability estimates.

**Behavioral test**	**Male h^2 ± se**	**Partner h^2 ± se**	**Strange h^2 ± se**
Time spent with partner	0.09 ± 0.10	0.16 ± 0.22	0.26 ± 0.27
Alloparental care before mating	0.08 ± 0.12	0.32 ± 0.26	0.13 ± 0.19
Alloparental care after mating	0.14 ± 0.17	0.31 ± 0.26	0.16 ± 0.23
Aggression towards a strange male before mating	0.03 ± 0.04	0.05 ± 0.09	0.19 ± 0.24
Aggression towards a strange male after mating	0.06 ± 0.11	0.07 ± 0.13	0.22 ± 0.28
Time in center before mating	0.30 ± 0.26	0.05 ± 0.10	0.26 ± 0.27
Time in center after mating	0.37 ± 0.18	0.04 ± 0.07	0.12 ± 0.16
